# Predictors of neurocognitive and psychological disorders in children after intensive care admission: A prospective cohort study

**DOI:** 10.1002/hsr2.1340

**Published:** 2023-06-15

**Authors:** Saptadi Yuliarto, Ardhanis Ramadhanti, Takhta Khalasha, Kurniawan Taufiq Kadafi, Ariani Ariani

**Affiliations:** ^1^ Department of Pediatrics, Medical Faculty Universitas Brawijaya, Saiful Anwar General Hospital Malang Indonesia; ^2^ Department of Pharmacology, Medical Faculty Universitas Brawijaya Malang Indonesia

**Keywords:** intensive care, neurocognitive disorders, pediatric, psychological disorders

## Abstract

**Background and Aims:**

Children admitted in the pediatric intensive care unit (PICU) often deal with long‐term morbidities affecting physical, cognitive, emotional, social, and psychiatric symptoms. We aimed to identify the internal and external factors which predict the occurrence of neurocognitive and psychological disorders in survivors at 3 months after PICU discharge.

**Methods:**

We identified 53 critically ill children, ages 4–18 years old, admitted in PICU for more than 24 h, and survived. We evaluated neurocognitive disorder with Pediatric Cerebral Perfomance Category (PCPC) and psychological disorders with Strengths and Difficulties Questionnaire (SDQ) at the time of PICU discharge and repeated in 3 months afterward. We evaluated the internal and external risk factors related to neurocognitive and psychological disorders in PICU survivors. The internal risk factors were age, gender, family composition, and socioeconomic status. The external risk factors were: surgical intervention, neurological disease, predicted death rate by pediatric index mortality (PIM)‐2 score, PICU length of stay (LOS), days of mechanical ventilation, and the number of therapeutic interventions.

**Results:**

There were significant improvement in neurocognitive disorders (p < 0.001), peer problems, (*p* = 0.01), and prosocial behaviors (*p* = 0.00) in children at 3 months after the PICU discharge. Age of 4–5 years has a significant effect on neurocognitive disorders (*p* = 0.04), while male gender (*p* = 0.02), low‐social economy, non‐intact family composition (*p* = 0.01), neurological disease (*p* = 0.04), surgical intervention (*p* = 0.01), and TISS score (*p* = 0.00) have a significant effect on psychological disorders in children at 3 months after the PICU discharge

**Conclusion:**

Neurocognitive disorders, peer problems, and prosocial behaviors improved in a few patients 3 months after PICU discharge. Age of 4–5 years was a risk factor of the persisted neurocognitive disorder, whereas male gender, low‐social economy, non‐intact family composition, neurological disease, surgical intervention, and TISS score were risk factors of persisted psychological disorder at 3 months after PICU.

## 1 INTRODUCTION

Post‐intensive care syndrome (PICS) is a disability acquired in patients who survive critical illness. This syndrome includes impaired physical, cognitive, or psychological functioning in patients undergoing treatment in the intensive care unit (ICU). PICS is defined as a new disorder or worsening of physical functioning (neuromuscular weakness post‐ICU treatment), neurocognitive (impaired thinking and consideration), or mental health status that occurs after a critical illness and persists after acute illness treatment.^1^ PICS in children is influenced by the patient's premorbid health state, neurological maturation, degree of family involvement, and processes of growth‐development, whereas it can affect children in several decades of life.^2^

PICS not only has implications for healthcare financing, but also causes long‐term limitations in children's life activities. New neurocognitive and psychological disorders can affect stages of development, school abilities, and social interactions, putting the child at risk of various problems arising later in life.^3^ However, most critically ill children do not receive consistent and comprehensive follow‐up care leading to early detection of cognitive impairment.^4^, ^5^ Systematic follow‐up care for children in PICU has not yet been developed. As a result, little is known about the recovery process of PICU survivors and their families, as well as the potential resources they need to achieve good outcomes. The focus of pediatric intensivists must shift from reducing mortality to enabling children to thrive beyond the PICU.^6^

Hospitalization in the PICU remains a life‐changing experience for children and their families, with potentially significant negative impacts on quality of life.^7^, ^8^ Age, resuscitation procedures, type and severity of disease, and length of stay in the intensive care unit are important factors affecting the quality of life of children after intensive care admission. Psychological disorders occur in 10–50% of post‐intensive care patients by showing new symptoms of depression, anxiety, posttraumatic stress disorder (PTSD), and sleep disorders.^9^ Approximately 30–80% of patients experience cognitive impairment post‐intensive care.^10^ Impairments occur in areas such as attention, executive function, memory, and processing speed, but there is little consensus as to which risk factors or combinations of risk factors lead to specific impairments.^11^, ^12^ Therefore, these sequelae need to be prevented since the early phase of intensive care and it is necessary to identify patients at high risk of developing PICS.^3^

In this study, children are followed at 3 months after PICU discharge to determine the influence of various risk factors during intensive care toward neurocognitive and psychological status in children. Identification of the risk factors may contribute to the treatment strategy of the intensive care to optimize post‐intensive care outcomes of patients, especially in the group of pediatric patients who are at risk population.

## 2 MATERIALS AND METHODS

### 2.1 Study design and participants

This study is a prospective cohort design. Consecutive sampling methods were conducted to recruit subjects that were children, aged 4–18 years who were survived and discharged following admission in the pediatric intensive care unit (PICU) of Saiful Anwar General Hospital, Malang, Indonesia for more than 24 h. The subjects were then followed up 3 months after the PICU discharge. Exclusion criteria were patients who were readmitted during the study period, or died during the 3 months after the PICU discharge, or missing data throughout the study period. The study was conducted from June 1, 2020, to June 1, 2021. Our PICU is a tertiary level, which provides 10 beds for medical and surgical cases, with 450‐600 cases annually.

### 2.2 Outcomes

The primary outcomes were neurocognitive disorders and psychological disorders at PICU discharge and 3 months after PICU discharge. The screening and enrolment of patients to the PICU was performed by research assistants. They screened patients in the PICU on a daily basis to identify eligible children. They collected all clinical study variables in a validated case report form. They also scheduled a follow‐up appointment with patients at discharge. At the time PICU discharge and 3 months afterward, children and their families were asked to complete health questionnaires: Pediatric Cerebral Performance Category (PCPC)^13^ and Strengths and Difficulties Questionnaire (SDQ).^14^

We also identified internal and external risk factors during intensive care toward neurocognitive and psychological status in PICU patients. The internal risk factors were age, gender, family composition, and socioeconomic status. The family composition was divided into intact (parents are married and live together with the children) and non‐intact (one of the parents does not live in the same house due to divorce or death). Based on the monthly income category set by Indonesian Central Bureau of Statistic, socioeconomic status was divided into low (monthly income <100 USD), middle (monthly income between 100 USD and 235 USD), and high (monthly income >235 USD).^15^ The external risk factors were surgical intervention, neurological disease, predicted death rate by pediatric index mortality (PIM)‐2 score, PICU length of stay (LOS), days of mechanical ventilation, and the number of therapeutic interventions.

### 2.3 Outcomes measures

The number of interventions evaluated using Therapeutic Intervention Scoring System (TISS) score (Supporting Information: Appendix 1).^16^ The neurocognitive disorder was evaluated by PCPC. PCPC scales are qualitative assessments of performance based on Glasgow Outcome Scale, whereas focuses on cognitive impairment.^13^ The psychological disorders were evaluated by Strengths and Difficulties Questionnaire (SDQ) at the time PICU discharge and 3 months afterward. SDQ is a brief, 25‐item, measure of behavioral and emotional difficulties that can be used to assess mental health problems in children.^14^ SDQ score consisted of four subscales of difficulties, that is emotional symptoms, conduct disorders, hyperactivity/attention deficit, and peer problems, as well as one subscale of strength, that is prosocial behavior. The total SDQ difficulty score is the summation of the scores of four difficulty subscales (excluding prosocial behavior subscales). The scale is expressed as normal, borderline, and abnormal categories.

### 2.4 Statistical analysis

The data were analyzed using IBM SPSS Statistics for Windows, version 25.0 software (IBM Corp.). Descriptive statistics are presented as numbers and proportions or medians, with 25%–75% interquartile ranges (IQRs). The baseline characteristics data were descriptively analyzed, then reported in median and IQR. Normality test performed to all parameters by Shapiro–Wilk test, followed by homogeneity test (Levene test). The comparison of PCPC and SDQ between intensive care discharge and 3 months afterward was performed by Dependent *T*‐Test or Wilcoxon signed rank test. All of the risk factors observed are being analyzed for the correlation with children's neurocognitive and psychological disorders. The bivariate association between each of the factors with PCPC and SDQ after intensive care is also being analyzed by Mann–Whitney Test, Kruskall–Wallis test, Pearson, or Spearman correlation test, based on the type of the data. Each factor which elicits significant differences on bivariate analysis is then analyzed using multivariate analysis (multiple logistic regression) to find the contributing factor. Two‐tailed tests were used, and *p* < 0.05 was regarded as statistically significant.

### 2.5 Ethical considerations

All the protocol in this study has already been approved by the Ethics Committee of Medical Faculty, Universitas Brawijaya, Malang, Indonesia (number of ethical approval letter: 400/291/K.3/302/2019), and written informed consent was obtained from all the participants or their parents.

## 3 RESULT

### 3.1 Subject characteristics

There were 53 subjects included in this study (Table 1), predominantly by males (62%) and age of 5–10 years (57%). Most of the family was intact and classified into middle social‐economic status. Most subjects were non‐surgical cases (72%) and non‐neurological disease (51%). The predicted death rate by PIM‐2 score was 15.96% (IQR: 3.7–22.2) and median PICU length of stay (LOS) was 5 (IQR: 2–16) days. TISS score median was 26 (IQR: 17–33) which indicated the various interventions during PICU admission.

**Table 1:** Baseline subjects characteristics.

Variable	Number (*n*=53)
*Internal factors*	
Gender, *n* (%)	
Male	33 (62)
Female	20 (38)
Age, *n* (%), years	
4–5	12 (22)
5–10	30 (57)
10–18	11 (21)
Family composition, *n* (%)	
Non‐intact	5 (9)
Intact	48 (91)
Socioeconomic status, *n* (%)	
Low	12 (23)
Middle	28 (53)
High	13 (24)
*External factors*	
Surgical intervention, *n* (%)	
Yes	15 (28)
No	38 (72)
Neurological disease, *n* (%)	
Yes	26 (49)
No	27 (51)
Predicted death rate by PIM‐2 score, %, median (IQR)	15.9 (3.7–22.2)
PICU length of stay, days, median (IQR)	5 (4.0–7.5)
Days of mechanical ventilation, days, median (IQR)	5 (2–16)
TISS score, median (IQR)	26 (17–33)

Abbreviations: IQR, interquartile range; PICU, pediatric intensive care unit; TISS, Therapeutic Intervention Scoring System.

### 3.2 Comparison of neurocognitive disorders between PICU discharge and 3 months afterward

**Figure 1:**
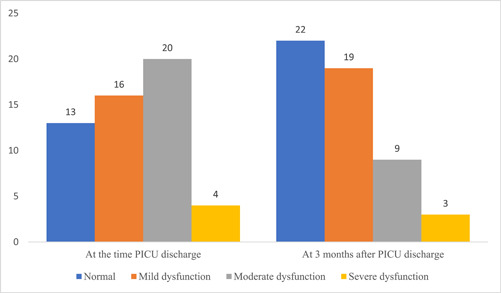
Neurocognitive status (PCPC scale) at the time PICU discharge and 3 months afterward. PICU, pediatric intensive care unit.

Twenty‐three subjects experienced improvement in neurocognitive disorders (9 mild to normal disabilities, 12 moderate to mild disabilities, and 2 subjects from severe to moderate disabilities) (*p* < 0.001) (Figure 1). This indicated a significant improvement of neurocognitive disorders in children 3 months after PICU discharge.

### 3.3 Comparison of psychological disorders between PICU discharge and 3 months afterward

#### 3.3.1 Total difficulties score

**Figure 2:**
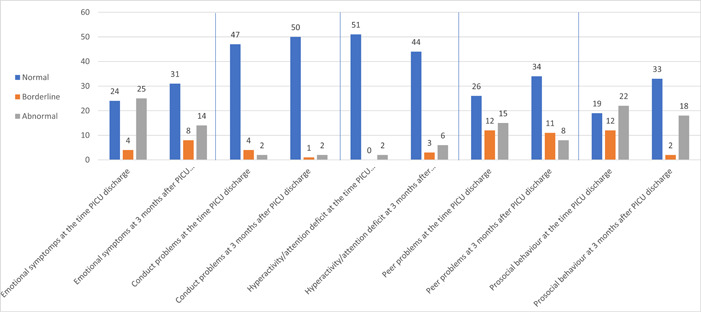
Psychological status (SDQ scale) at the time PICU discharge and 3 months afterward. PICU, pediatric intensive care unit.

Nineteen subjects improved of psychological status (13 borderlines to normal, 4 abnormal to normal, and 2 subjects abnormal to borderline) (Figure 2). However, there was no difference of total difficulties score at PICU discharge and 3 months afterward (*p* = 0.08).

#### 3.3.2 Emotional symptoms

Twenty‐one subjects experienced an improvement in emotional symptoms (3 borderline to normal, 11 abnormal to normal, and 7 subjects abnormal to borderline). Meanwhile, as many as eight subjects worsened (seven normal to one borderline and six abnormal, and one subject borderline to abnormal) (Figure 2). There was no difference of emotional symptoms between PICU discharge and 3 months afterward (*p* = 0.08).

#### 3.3.3 Conduct disorders

Five subjects experienced improvement in conduct disorders (three borderline to normal and two subjects abnormal to normal). Meanwhile, as many as two patients worsened (normal to abnormal) (Figure 2). There was no difference of conduct disorders between PICU discharge and 3 months afterward (*p* = 0.60).

#### 3.3.4 Hyperactivity/attention deficit

Only one subject improved hyperactivity/attention deficit disorder (abnormal to borderline), while as many as seven subjects worsened (two normal to borderline, five subjects normal to abnormal) (Figure 2) (*p* = 0.02). It indicated a new condition of hyperactivity/attention deficit disorder in children 3 months PICU admission.

#### 3.3.5 Peer problems

Fifteen subjects improved of peer problem (seven borderline to normal, 4 abnormal to normal, and 4 subjects abnormal to borderline) (Figure 2) (*p* = 0.01), which indicated significant improvement of peer problems in children 3 months after PICU admission.

#### 3.3.6 Prosocial behaviors

Fourteen subjects improved prosocial behavior (10 borderlines to normal and 4 subjects abnormal to normal) (Figure 2) (*p* < 0.001), which indicated significant improvement of prosocial behavior in children 3 months post‐intensive care.

### 3.4 Risk factor of neurocognitive disorders

As seen in Table 2, the age (4–5 years) was a risk of neurocognitive disorder at 3 months after PICU discharge (OR: 12.18 (1.66–89.75), *p* = 0.04), whereas ages of 5–10 years, surgery intervention, PIM score, PICU LOS, days of mechanical ventilation, and TISS score were not correlated with that condition.

**Table 2:** Multivariate analysis of risk factors for neurocognitive disorders 3 months after PICU discharge.

Risk factors	OR (95% CI)	*p*
Age (4–5 years old)	12.18 (1.66–89.75)	0.01^a^
Age (5–10 years old)	5.55 (1.01–30.63)	0.05
Surgical intervention	1.57 (0.42–5.88)	0.50
PIM score^b^	1.02 (0.97–1.08)	0.42
PICU length of stay	1.23 (0.93–1.63)	0.14
Days of mechanical ventilation	1.19 (0.82–1.75)	0.38
TISS score^c^	1.03 (0.94–1.15)	0.51

Abbreviations: PICU, pediatric intensive care unit; PIM, pediatric index mortality; TISS, Therapeutic Intervention Scoring System.

^a^ All correlation were significant at *p* < 0.05.

^b^ Increased PIM score.

^c^ Increased TISS score.

### 3.5 Risk factor of psychological disorders

As seen in Table 3, only six risk factors increase the probability of having psychological disorder at 3 months after PICU discharge, that is: non‐intact family composition, neurological disease, and surgical intervention which led to the peer problem (OR: 18.17 [1.98–166.83], *p* = 0.01; OR: 5.03 [1.11–22.81], *p* = 0.04; and OR: 6.46 [1.69–24.66], *p* = 0.01, respectively), male gender and low social economy which led to hyperactivity/attention deficit problems (OR: 33.08 [1.58–692.98], *p* = 0.02 and OR 33.58 [1.95–579.4], *p* = 0.02, respectively), and TISS score which led to the emotional symptoms (OR: 1.49 [1.20–1.85], *p* < 0.001). In addition, neurological disease was negatively associated with emotional symptoms (OR 0.01 [0.00 = 0.31], *p* = 0.01).

**Table 3:** Multivariate analysis of risk factor to psychological disorders.

Risk factors	OR (95% CI)	*p*
*Total difficulties score of SDQ*		
Social economy status (low)	13.57 (0.53–348.28)	0.12
Social economy status (middle)	6.42 (0.38–107.88)	0.19
Neurological disease	0.72 (0.08–6.32)	0.77
Surgical intervention	1.87 (0.23–15.29)	0.56
PICU length of stay	1.04 (0.74–1.48)	0.81
Days of mechanical ventilation	1.30 (0.78–2.19)	0.32
PIM score	1.04 (0.96–1.13)	0.29
TISS score	1.11 (0.95–1.29)	0.18
*Emotional symptoms*		
Age (4–5 years old)	44.08 (0.78–2507.40)	0.07
Age (5–10 years old)	1.67 (0.14–19.45)	0.68
Male	0.91 (0.15–5.61)	0.92
Social economy status (low)	19.61 (0.47–813.22)	0.12
Social economy status (middle)	0.75 (0.06–9.78)	0.82
Neurological disease	0.01 (0.00–0.31)	0.01^a^
PICU length of stay	1.28 (0.83–1.95)	0.27
Days of mechanical ventilation	1.09 (0.63–1.90)	0.76
PIM score^b^	1.01 (0.94–1.09)	0.76
TISS score^c^	1.49 (1.20–1.85)	<0.001^a^
*Conduct disorders*		
Surgical intervention	1.35 (0.019–97.81)	0.89
PICU length of stay	1.47 (0.58–3.70)	0.42
Days of mechanical ventilation	0.67 (0.19–2.37)	0.53
PIM score	0.84 (0.48–1.44)	0.52
TISS score	4.71 (0.23–94.35)	0.31
*Hyperactivity/attention deficit*		
Male	33.08 (1.58–692.98)	0.02^a^
Social economy status (low)	33.58 (1.95–579.40)	0.02^a^
Social economy status (middle)	0.72 (0.04–12.06)	0.82
PIM score^b^	1.06 (0.98–1.14)	0.17
Days of mechanical ventilation	0.90 (0.66–1.23)	0.51
TISS score^c^	1.11 (0.95–1.29)	0.20
*Peer problems*		
Family composition (non‐intact)	18.17 (1.98–166.83)	0.01^a^
Neurological disease	5.03 (1.11–22.81)	0.04^a^
Surgical intervention	6.46 (1.69–24.66)	0.01^a^
*Prosocial behavior*		
Age (4–5 years old)	4.45 (0.44–44.70)	0.20
Age (5–10 years old)	1.45 (0.19–11.34)	0.72
Male	4.79 (0.78–29.49)	0.09
Neurological disease	7.45 (0.99– 55.87)	0.05
Surgical intervention	1.14 (0.16–8.17)	0.90
PICU length of stay	1.18 (0.84–1.64)	0.34
Days of mechanical ventilation	0.87 (0.53–1.42)	0.57
PIM score^b^	1.03 (0.96–1.12)	0.39
TISS score^c^	1.12 (0.97–1.29)	0.13

Abbreviations: PICU, pediatric intensive care unit; PIM, pediatric index mortality; TISS, Therapeutic Intervention Scoring System.

^a^ Correlation was significant.

^b^ Increased PIM score.

^c^ Increased TISS score.

## 4 DISCUSSION

In this prospective observational cohort study, we found that 43% (23/53) subjects who had neurocognitive disorder at PICU discharge improved at 3 months afterward. This result is consistent with previous studies that showed cognitive function of ICU patients mostly recovered within the first 3 months to 12 months.^17^, ^18^ The cognitive function tests revealed a significant impairment in executive function and general intellectual competence at 3 months, but there is clear evidence of a return towards more “normal” cognitive ability by 9 months.^19^ Long‐term follow‐up and rehabilitation program to detect, support, and treat PICU survivor having neurocognitive and psychological problems may support daily‐life activities and minimize the impact on children's well‐being and future development.^20^, ^21^

The exact pathophysiology of neurocognitive disorders due to intensive care is unrevealed, but it is suggested caused by brain dysfunction during acute processes and during intensive care.^22^, ^23^ Many external factors may play a significant role in influencing the neurocognitive condition of the child after intensive care. The prior study mentioned that patients with neurological diseases often have moderate neurocognitive disorder, due to the direct involvement of the central nervous system.^2^ The last two studies also reported a positive association between disease severity (high PIM score) and an increase in PCPC scores. Some invasive interventions, as well as, types of cardiovascular, neurological, and gastrointestinal diseases requiring surgery are also the influencing factors.^19^

We found that some subjects improved from their psychological domain, that is peer problems and prosocial behaviors at 3 months after PICU discharge. In contrast, one study reported an improvement in levels of psychological distress after long‐term periods.^24^ Similarly, another study showed that eight patients still developed psychological distress at 12‐month follow‐up.^25^ However, we also found that even though many of PICU survivors report an improvement in psychological distress at 3 months after PICU discharge, some subjects report worsened in psychological distress; hence, our results indicate that the psychological outcome after PICU discharge may differ considerably on an individual level. Therefore, screening for PICS during follow‐up of PICU discharge could help identify high‐risk patients who need psychological support.

Our study showed age of 4–5 years was the sole risk factor of the persisted neurocognitive disorder at 3 months after PICU discharge. Similar to our findings, another study found that children with neurocognitive disorders after PICU admission are significantly younger than those without neurocognitive disorders.^26^ One theory of cognitive development in children states that the age of 2–5 years is a critical stage of cognitive development including attention, short‐ and long‐term memory. Disturbances at this stage will result in disturbance of cognitive development. Critical illness in children that occurs during the growth and development process, mainly at a younger age stage, lead to more severe neurocognitive impact.^2^, ^27^ Therefore, a younger age child at PICU admission is associated with lower cognitive function.

In our study, the persisted psychological disorder, especially in the aspect of emotional, hyperactivity/attention deficit, and peer problems at 3 months after PICU discharge, were influenced by some risk factors, that is, male gender, low socioeconomy, non‐intact family composition, neurological disease, surgical intervention, and TISS score. Currently, it is still lack of reference which correlated gender and psychological disorders in post‐intensive care children. One study mentioned that boys are more likely to show symptoms of hyperactivity and perform impulsive actions than women, with the prevalence ratio of boys to girls were 2–3:1.^28^

The influence of socioeconomic status on children's psychological conditions was also reported in one study, revealing that low socioeconomic status increases the risk of behavioral, emotional, and hyperactivity disorders in children. Low socioeconomic status is often accompanied by a low level of parental education, which also plays a role in the emergence of children's psychological disorders.^29^ Increases in family income are associated with a corresponding increase in child physical health, behavioral health, development, and healthcare access and utilization.^30^ This is in accordance with our finding, which is low socioeconomic status increases the risk of hyperactivity/attention deficit disorder of children 3 months after intensive care. Therefore, low socioeconomic status is associated with adverse mental health outcomes.

Another study reported family composition also plays an important role in the overall condition, not only the psychological of the child. The composition of the intact family provides stability in the process of child development, including the psychological state.^31^ The process of restoring the child's condition after intensive care will be supported by the intact family. This is in accordance with our finding which shows that the composition of the family which is not intact could increase the chances of psychological disorders in children. Our results suggest a decreased risk of incidents of psychological disorders in intact families associated with abundance of family visitation during a patient's stay in the PICU as a protective factor for the occurrence of depression, anxiety, and posttraumatic stress disorders after PICU discharge.^32^

Types of diseases experienced by children also affect the psychological outcomes of pediatric patients. Chronic diseases that require various types of therapy, in particular neurological diseases, negatively affect the psychological status of the child. It was shown that children with neurological diseases may lead to the emotional disorder.^33^ We were unable to analyze differences in psychological status in children admitted with different diseases due to the small sample size of the study. Possibly, future research should analyze other risk factors such as brain trauma and pathophysiologic mechanisms mediating psychological dysfunction in larger populations.

In our study, surgical procedures could impact peer and prosocial behavior disorders. This condition mainly occurs in children who require long admission after operative procedures. The anatomical and functional changes of the body, as well as the need for long‐term care, are suggested to affect children's interactions. Furthermore, children who are particularly exposed to high numbers of invasive procedures appear to be at increased risk for developing negative psychological sequelae.^34^ Awareness of long‐term sequelae may result in supportive programs such as psychosocial care interventions, arranging referrals to adequate mental health‐care professional, which showed importance by both parents and children during an extremely stressful period of their life.^35^

Previous studies have reported longer lengths of PICU stays and more severe diseases could influence the emergence of psychological disorders.^36^ In a recent meta‐analysis, neither illness severity at admission nor length of PICU stay were risk factors for subsequent cognitive morbidity.^37^ The PICU survivors were at a higher risk of posttraumatic stress disorders (PTSD) than other patients, and the number of invasive procedures during PICU admissions has also caused traumatic experiences in children.^36^, ^38^ In this study, degree of intervention, showed by an increase in TISS scores, had a significant effect on children's emotional disorders. Children that needed more aggressive treatment in the PICU probably had a pre‐existing severe illness, and this is probably the more important indicator in determining functional outcome.

There are some limitations in our study. First, the study was single‐center study, so the subjects were homogeneous in social and cultural background. Second, some aspects, which potentially affect patient outcomes (including post‐intensive care interventions, parental education level, race/ethnicity, religion, and parental involvement in post‐intensive care), were not analyzed. Further research is needed involving a larger sub.

## 5 CONCLUSION

Neurocognitive disorders, peer, and prosocial problems improved in a few patients 3 months after PICU discharge. Age of 4–5 years was a risk factor for the persisted neurocognitive disorder, whereas male gender, low‐social economy, non‐intact family composition, neurological disease, surgical intervention, and TISS score were risk factors of persisted psychological disorder at 3 months after PICU.

## AUTHOR CONTRIBUTIONS

**Saptadi Yuliarto**: Conceptualization; writing—review & editing. **Ardhanis Ramadhanti**: Investigation; writing—original draft. **Takhta Khalasha**: Writing—review & editing. **Kurniawan Taufiq Kadafi**: Formal analysis; investigation. **Ariani Ariani**: Formal analysis; investigation. All authors have read and approved the final version of the manuscript.

## CONFLICT OF INTEREST STATEMENT

The authors declare no conflict of interest.

## TRANSPARENCY STATEMENT

The lead author Saptadi Yuliarto affirms that this manuscript is an honest, accurate, and transparent account of the study being reported; that no important aspects of the study have been omitted; and that any discrepancies from the study as planned (and, if relevant, registered) have been explained.

## Supporting information

Supporting information.

## Data Availability

The authors confirm that the data supporting the findings of this study are available within the article and its supplementary material. Raw data that support the findings of this study are available from the corresponding author, upon reasonable request. Saptadi Yuliarto had full access to all of the data in this study and takes complete responsibility for the integrity of the data and the accuracy of the data analysis.
