# Supplementary material for: Predictors of neurocognitive and psychological disorders in children after intensive care admission: A prospective cohort study
Source: Health Sci Rep. 2023 Jun 15;6(6):e1340. doi: 10.1002/hsr2.1340 (PMC10268591; doi:10.1002/hsr2.1340)
Supplement: Supplementary file 1 — Supporting information. [file HSR2-6-e1340-s001.docx]

**APPENDIX**

**Appendix 1. Therapeutic Intervention Scoring System (TISS-28)**

| Category | TISS-28 Points |
| --- | --- |
| Basic Activities  Standard Monitoring (hourly vital signs, fluid balance)  Biochemical and Microbiological Investigation  Single Medication (any route)  Multiple intravenous medications  Care and prevention of decubitus and daily dressing changes  Frequent dressing changes (at least one time per each nursing shift)  Care of drains | 5  1  2  3  1  1  3 |
| Cardiovascular Support  Single vasoactive medication  Multiple vasoactive medications  Intravenous replacement of large fluid losses (> 3L/m^2^/day)  Peripheral artery catheter  Pulmonary artery flotation catheter  Central venous line  Cardiopulmonary Resuscitation after arrest in the past 24 hours | 3  4  4  5  8  2  3 |
| Specific intervention  Single specific interventions in the ICU (naso or orotracheal intubation, cardioversion,  introduction of pacemaker, endoscopies, emergency surgery in the past 24 hours)  Multiple specific interventions in the ICU (more than one described above)  Specific interventions outside ICU (surgery or diagnostic procedures) | 3  5  5 |
| Ventilatory Support  Mechanical ventilation  Supplementary ventilation support (supplementary oxygen by any method except if  mechanical ventilation parameters apply)  Care of artificial airways (endotracheal tube or tracheostoma)  Treatment for improving lung function (e.g. thorax physiotherapy, incentive spirometry,  inhalation therapy, intratracheal suctioning) | 5  2  1  1 |
| Renal support  Hemofiltration/ dialytic techniques  Quantitative urine output measurement  Active diuresis (e.g. furosemide > 0.5mg/Kg/day) | 3  2  3 |
| Neurologic support  Measurement of intracranial pressure | 4 |
| Metabolic support  Treatment of complicated metabolic acidosis/alkalosis  Intravenous alimentation  Enteral feeding through gastric tube or other route (e.g. jejunostomy) | 4  3  2 |

**Appendix 2. . Pediatric Cerebral Perfomance Category (PCPC)**

| Score | Category | Clinical feature |
| --- | --- | --- |
| 1 | Normal | - Normal at age appropriate level - School age child attends regular school classroom |
| 2 | Mild disability | - Conscious alert and able to interact at an age appropriate level - School age child attending regular school classroom but grade perhaps not appropriate for age - May have a mild neurologic deficit |
| 3 | Moderate disability | - Conscious - Sufficient cerebral function for age-appropriate independent activities of daily life - School age child attending special education classroom - May have learning deficit |
| 4 | Severe disability | - Conscious - Dependent on others for daily support because of impaired brain function |
| 5 | Coma or vegetative state | - Any degree of coma without any of the criteria for brain death - Unawareness even if awake in appearance without interaction with the environment - Cerebral unresponsiveness - No evidence of cortical function and not aroused by verbal stimuli - Possibly some reflexive responses spontaneous eye opening and/or sleep-wake cycles |
| 6 | Brain death | - Apnea OR - Areflexia OR - Electroencephalographic (EEG) silence |

**Appendix 3. Strength and Difficulties Questionnaire (SDQ)**

| **Items of SDQs scale** | **Not True** | **Somewhat True** | **Certainly True** |
| --- | --- | --- | --- |
| **Emotional problems scale** |  |  |  |
| Item 3 : Often complains headache (I get a lot of headaches) | 0 | 1 | 2 |
| Item 8 : Many worries (I worry a lot) | 0 | 1 | 2 |
| Item 13 : Often unhappy, downhearted (I am often unhappy) | 0 | 1 | 2 |
| Item 16 : Nervous or clingy in new situations (I am nervous in new situations) | 0 | 1 | 2 |
| Item 24 : Many fears, easily scared (I have many fears) | 0 | 1 | 2 |
| **Conduct problems scale** |  |  |  |
| Item 5 : Often has temper tantrums or hot tempers (I get very angry) | 0 | 1 | 2 |
| Item 7 : Generally obedient (I usually do as I am told) | 2 | 1 | 0 |
| Item 12 : Often fight with other child (I fight a lot) | 0 | 1 | 2 |
| Item 18 : Often lies or cheats (I am often accused of lying or cheating) | 0 | 1 | 2 |
| Item 22 : Steals from home, school, or elsewhere (I take things that are not mine) | 0 | 1 | 2 |
| **Hyperactivity/attention deficit scale** |  |  |  |
| Item 2 : restless,overactive (I am restless) | 0 | 1 | 2 |
| Item 10 : Constantly fidgeting or squirming (I am constantly fidgeting) | 0 | 1 | 2 |
| Item 15 : Easily distracted, concentration wanders (I am easily distracted) | 0 | 1 | 2 |
| Item 21 : Thinks things out before acting (I think before I do things) | 2 | 1 | 0 |
| Item 25 : Sees tasks through to the end (I finish the work I am doing) | 2 | 1 | 0 |
| **Peer problems scale** |  |  |  |
| Item 6 : Rather solidarity, tends to play alone (I am usually on my own) | 0 | 1 | 2 |
| Item 11 : Has at least one good friend (I have one goof friend or more) | 2 | 1 | 0 |
| Item 14 : Generally liked by other children (Other people my age generally like me) | 2 | 1 | 0 |
| Item 19 : Picked on or bullied by other children (Other children or young people  pick on me | 0 | 1 | 2 |
| Item 23 : Gets on better with adults than with other children (I get on better with  adults than with people my age) | 0 | 1 | 2 |
| **Prosocial scale** |  |  |  |
| Item 1 : Considerate of other people’s feelings (I try to be nice to other people) | 0 | 1 | 2 |
| Item 4 : Shares readily with other children (I usually share with others) | 0 | 1 | 2 |
| Item 9 : Helpful if someone is hurt (I am helpful if someone is hurt) | 0 | 1 | 2 |
| Item 17 : Kind to younger children (I am kind to younger children) | 0 | 1 | 2 |
| Item 20 : Often volunteers to help other (I often volunteer to help others) | 0 | 1 | 2 |
